# An improved spinning lens test to determine the stiffness of the human lens

**DOI:** 10.1016/j.exer.2010.10.010

**Published:** 2011-01

**Authors:** H.J. Burd, G.S. Wilde, S.J. Judge

**Affiliations:** a Department of Engineering Science, University of Oxford, Parks Road, Oxford, OX1 3PJ, UK; b Department of Physiology, Anatomy and Genetics, University of Oxford, OX1 3QX, UK

**Keywords:** lens, stiffness, presbyopia, finite element

## Abstract

It is widely accepted that age-related changes in lens stiffness are significant for the development of presbyopia. However, precise details on the relative importance of age-related changes in the stiffness of the lens, in comparison with other potential mechanisms for the development of presbyopia, have not yet been established. One contributing factor to this uncertainty is the paucity and variability of experimental data on lens stiffness. The available published data generally indicate that stiffness varies spatially within the lens and that stiffness parameters tend to increase with age. However, considerable differences exist between these published data sets, both qualitatively and quantitatively. The current paper describes new and improved methods, based on the spinning lens approach pioneered by Fisher, R.F. (1971) ‘The elastic constants of the human lens’, Journal of Physiology, 212, 147–180, to make measurements on the stiffness of the human lens. These new procedures have been developed in an attempt to eliminate, or at least substantially reduce, various systematic errors in Fisher’s original experiment. An improved test rig has been constructed and a new modelling procedure for determining lens stiffness parameters from observations made during the test has been devised. The experiment involves mounting a human lens on a vertical rotor so that the lens spins on its optical axis (typically at 1000 rpm). An automatic imaging system is used to capture the outline of the lens, while it is rotating, at pre-determined angular orientations. These images are used to quantify the deformations developed in the lens as a consequence of the centripetal forces induced by the rotation. Lens stiffness is inferred using axisymmetric finite element inverse analysis in which a nearly-incompressible neo-Hookean constitutive model is used to represent the mechanics of the lens. A numerical optimisation procedure is used to determine the stiffness parameters that provide a best fit between the finite element model and the experimental data. Sample results are presented for a human lens of age 33 years.

## Research highlights

► A new spinning lens rig has been devised to measure the stiffness of the human lens. ► Finite element analysis procedures are developed to determine lens stiffness data from the test results. ► The experiment is capable of investigating spatial stiffness variations in the lens.

## 1 Introduction

### 1.1 Accommodation, presbyopia and lens stiffness

The human lens is suspended within the globe of the human eye by a set of zonular fibres, that connect the lens to the ciliary muscle. When the normal young eye views a distant object, the ciliary muscle is relaxed and tension in the zonules is maximal. In this state the eye is said to be unaccommodated. To bring a nearby object into focus, the ciliary muscle contracts with the consequence that the tension in the zonules reduces and the lens adopts a thicker and more rounded shape. The deformations that develop in the lens during this process cause the optical power of the eye to increase. In this state the eye is said to be accommodated. It is well known that the effectiveness of this accommodation mechanism decreases with increasing age and that, from middle-age onwards, the accommodation range is minimal. This limiting condition is known as presbyopia.

In recent years there has been considerable interest in the development of new forms of surgical intervention to restore some measure of accommodation to presbyopes. A detailed understanding of the mechanical performance of the individual components of the accommodation apparatus would be of assistance in assessing and optimising these proposed interventions. Also, at a more fundamental level, there is continued interest in developing a more secure understanding of the natural ageing processes in the human eye and the way in which these processes lead to the development of presbyopia.

Although presbyopia has been attributed in various ways to different ageing mechanisms, e.g. Charman (2008), it is generally assumed that age-related changes in the stiffness of the lens are a significant contributing factor. To test this assumption, robust numerical data are needed on lens stiffness, and on the way in which the stiffness develops with age. Current published experimental data on lens stiffness (e.g. Fisher, 1971; Heys et al., 2004; Hollman et al., 2007; Weeber et al., 2007) indicate that the lens becomes stiffer with age. However, significant differences exist between the numerical values of lens stiffness data reported in the literature. This lack of uniformity in the published data reflects, to varying degrees, the difficulties in obtaining high quality post-mortem lenses, sample swelling and damage during transport and storage (e.g. see Augusteyn et al., 2006), technical challenges associated with working at a small scale and with soft materials, systematic errors associated with the various test procedures that have been adopted and genuine biological variations between samples obtained from different donors.

### 1.2 Mechanics of the lens

The lens is a complex structure. It consists of an intricate arrangement of specialised cells, known as lens fibres, enclosed within a thin and relatively stiff collagen-rich nearly acellular membrane known as the capsule. The internal, cellular, regions of the lens (referred to in this paper as the lens substance) and the external capsule are distinct biological structures. It is therefore convenient to treat them separately for the purpose of developing an understanding of the mechanical characteristics of the lens. Certain features of the lens substance are known to vary spatially within it. It is currently understood, for example, that the stiffness of the lens substance (e.g. Heys et al., 2004; Weeber et al., 2007) and the refractive index (e.g. Navarro et al., 2007; Kasthurirangan et al., 2008) both vary with position. The lens substance is also known to be non-homogeneous in a structural sense. The nucleus, for example, is a central portion of the lens that is generally regarded as being distinct from the surrounding cortex (e.g. Augusteyn, 2007, 2010).

Previous experimental studies on the mechanics of the lens substance have mostly proceeded on the basis that it can be represented by an isotropic linear elastic constitutive model. (A constitutive model is a mathematical framework to represent the mechanical behaviour of a material). The non-homogeneous nature of the lens substance is treated by allowing the stiffness parameters to vary with position. The use of isotropic linear elasticity is arguably the simplest constitutive model that can sensibly be employed in this context. It requires the specification of only two independent material parameters; for the purpose of modelling the lens it is convenient to choose the shear modulus *G* and the bulk modulus *K* as these parameters. It is typically assumed in the interpretation of data from experimental studies that the lens substance is incompressible; this has the consequence that *K* = ∞. (In practice, to avoid numerical difficulties in a finite element analysis, the bulk modulus is normally set to a large but finite value.) As a consequence, only one parameter, the shear modulus *G*, is needed to characterise the stiffness of the lens. This view of the lens substance as an incompressible material is supported by MRI studies that indicate that total lens volume is conserved during accommodation (e.g. Hermans et al., 2009). (It should be noted, of course, that although MRI studies may indicate that the total volume of the lens is conserved during accommodation, they do not demonstrate the more restrictive condition that each individual material point in the lens behaves in an incompressible way. It is plausible, for example, that fluid flow may occur within the lens as a result of the stress gradients set up by the accommodation process. This flow would cause the volume of some parts of the lens to reduce and other parts to increase but, on the basis that water is assumed to be effectively incompressible, the total lens volume is conserved.)

Although linear isotropic elasticity provides a convenient approach, it is important to note the possibility that the behaviour of the lens substance may depart significantly from this rather simple model. For example, the highly organised and directional structure of the lens fibres (e.g. Taylor et al., 1996) suggest that the lens substance may behave in an anisotropic manner. Also, dynamic mechanical analysis (DMA) data from Weeber et al. (2005) indicate that the lens exhibits time-dependent behaviour (a feature that is not captured in linear elasticity). Any time-dependency in the lens might derive from visco-elastic behaviour inherent in the molecular structures of the various proteins within the lens. Alternatively (or perhaps in addition) it may arise as a consequence of localised fluid flow within the lens in response to gradients of stress. In spite of these open questions, it is considered that the relative simplicity of the isotropic elasticity approach means that it provides a useful and robust framework within which to describe the mechanics of the lens substance.

Conventional linear elastic theory is formulated in the framework of linear continuum mechanics. It is generally understood, however, that geometric non-linear effects associated with finite displacements may need to be included within any computational model of the mechanics of the lens to achieve realistic results (e.g. Burd et al., 1999). Although linear elasticity is capable of being embedded within a geometric non-linear computational framework, this leads to procedures that lack the rigorous theoretical basis of mathematical formulations that are based on non-linear continuum mechanics and developed within the framework of hyperelasticity (e.g. Bonet and Wood, 1997; Holzapfel, 2000). In view of this, a non-linear continuum mechanics hyperelastic approach is adopted in the finite element analyses described later in this paper. Geometric non-linear effects are captured, in a rigorous way, in this approach. The finite element analysis is based on the neo-Hookean constitutive model to represent the mechanics of the lens. This is a relatively simple hyperelastic model that may be specified in terms of a shear modulus and a bulk modulus that, for small strains, provide equivalence with parameters of the same name that are conventionally adopted in linear elasticity theory.

### 1.3 Spinning lens concept

The purpose of this paper is to describe an experimental method, based on the spinning lens concept devised by Fisher (1971), to determine numerical values of parameters to describe the stiffness of the human lens substance. One of the principal applications of the data is in the development of numerical models (e.g. based on the finite element method) of the natural accommodation process (e.g. Burd et al., 2002; Weeber and van der Heijde, 2007). The data may also be useful to assist in the assessment of the likely effectiveness of proposed surgical treatments for presbyopia, particularly those that involve modifications to the mechanical characteristics of the lens substance (e.g. Schumacher et al., 2009).

In Fisher’s original experiment, the lens was spun on its optical axis and the cross-section of the spinning lens was imaged using flash photography. The observed changes in axial thickness and equatorial diameter were used, in conjunction with an approximate analytical model of the deformations induced in the spinning lens, to infer values of lens substance Young’s modulus (which is closely related to the shear modulus). Fisher (1971) presented data on a total of 40 lenses, in the age range 0–67 years. A study by Burd et al. (2006) raised various questions about possible systematic errors in the procedures adopted by Fisher. For example, the analysis of the data given by Fisher (1971) ignored the constraining effect of the capsule whereas it seems implausible that the mechanical effect of the capsule can be neglected in this way without introducing systematic errors in the computed stiffness data.

It is suggested that some, if not all, of the systematic errors in the Fisher (1971) test (as identified in Burd et al., 2006) can be eliminated or substantially reduced by improved experimental design and data processing. The new version of the spinning lens test described in this paper has been developed with this specific aim. A key feature of the proposed procedure is that data on the stiffness of the lens substance are determined on the basis of testing a lens that has had its capsule carefully removed. This has the very substantial advantage over the Fisher (1971) test in that the experiment is conducted solely on the lens substance and not the combined system of lens capsule and lens substance. The analysis of the experimental data can therefore proceed without the need to introduce assumptions on the mechanical influence of the capsule. Removal of the capsule does, of course, lead to the possibility of damage to the external lens fibres and increased moisture loss during the test. The authors have concluded, however, that the detrimental effect of capsule removal (which can in any case be minimised by careful experimental design and procedure) is out-weighed by the very considerable benefits of being able to conduct tests on the isolated lens substance.

The spinning lens test has the considerable merit that the lens tissue is subject to relatively minor mechanical disturbance prior to and during testing. This is in contrast to the use of indentation testing, (Heys et al., 2004; Weeber et al., 2007) in which the lens is cut to allow internal stiffness variations to be investigated. This cutting process may disturb the structure of the lens fibres. However, it should be noted that the spinning lens test does not allow the spatial variation of lens stiffness to be determined directly. Instead, it is necessary to infer the internal variation of stiffness from measurements on the geometry of the lens outline. This requires the use of a parametric model for the spatial variation of stiffness within the lens, together with an optimisation procedure to determine the values of the parameters that provide a best fit between the observed lens outline and the outline computed using the model.

The experimental procedures described in this paper have been used to complete a programme of tests on human lenses. One of the lenses from this test programme, from a donor of age 33 years, is adopted in the paper to illustrate the experimental methods and associated data analysis procedures. Stiffness data on the other lenses tested during this study will be the subject of a future publication.

## 2 Materials and methods

### 2.1 Spinning lens rig design

In this original version of the test (Fisher, 1971), the imaging system was not synchronised with the angular position of the lens. This meant that the photographs of the lens cross-section were obtained at random angular orientations of the lens. The equatorial diameter and axial thickness changes induced by the rotational motion were therefore based on the average of values determined in a non-systematic way from lenses imaged at these random orientations. An alternative system is adopted in the experimental arrangement described in the current paper in which the imaging system is synchronised with the angular position of the lens. This allows the profile of the spinning lens to be imaged at pre-determined and equally spaced angular orientations that are independent of the rotational speed. This procedure means that the shape of the rotating lens (as determined from the lens cross-section images) can be related to the initial shape of the lens cross-section when viewed from the same angular orientation. Any departures from rotational symmetry of the lens do not therefore induce systematic errors in the analysis of the data.

**Fig. 1:**
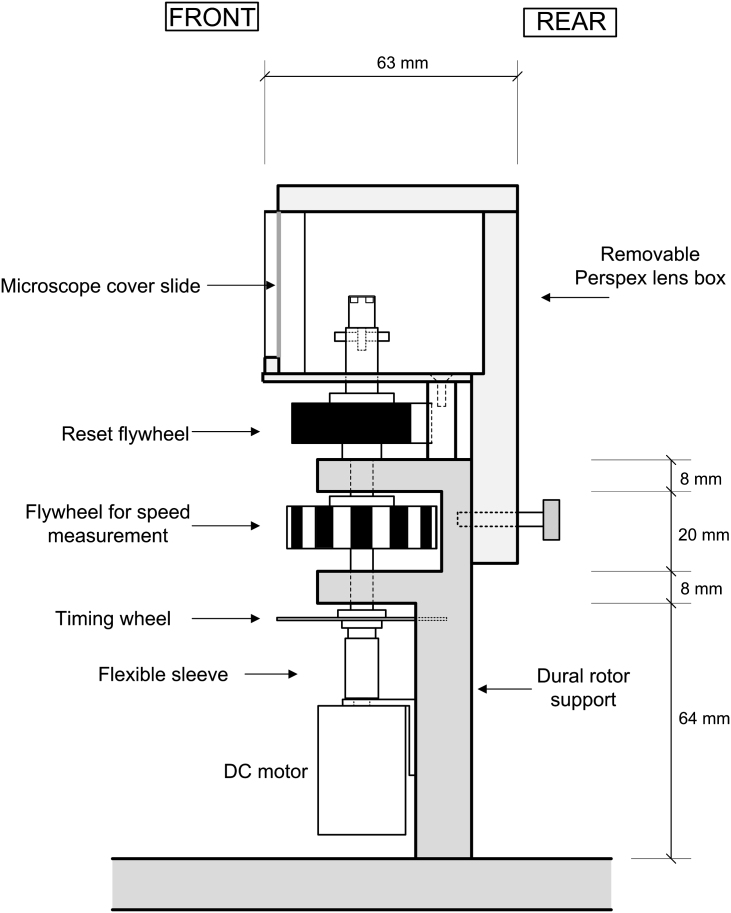
Side view of the spinning lens rig drawn to scale. The three optical sensors are omitted for clarity.

The test rig is illustrated in Fig. 1. The principal component of the rig is a vertical rotor held in two sets of bearings by a rotor support structure machined from Dural^®^. It is driven by a direct current (DC) motor (Maxon A-Max 22 mm diameter) connected to the rotor by a flexible sleeve. The rotational speed of the rotor is adjusted manually via a variable voltage supply.

Two brass flywheels are mounted on the shaft; the inertia provided by these flywheels ensures that the rotational speed remains steady. The lower flywheel (indicated as ‘flywheel for speed measurement’ on Fig. 1) is used to measure the rotational speed. It is painted alternately with 12 black and 12 white stripes of equal width; these stripes are accurately defined by vertical lines machined onto the flywheel. The stripes are detected by an optical reflection sensor (Honeywell HOA0708, omitted from Fig. 1 for clarity). When the flywheel rotates, the sensor produces a square wave signal which is used, via a digital counter, to determine the rotational speed (to a precision of 5 rpm). One-half of the perimeter of the upper flywheel (termed ‘reset flywheel’ on Fig. 1) is painted black and the other half is painted white. Vertical lines machined onto the perimeter define the edges of each painted zone. The edge between the zones is detected by another optical reflection sensor (Honeywell HOA0708, omitted from Fig. 1); this signal is used to reset the imaging system counter (as described in Section 2.3).

**Fig. 2:**
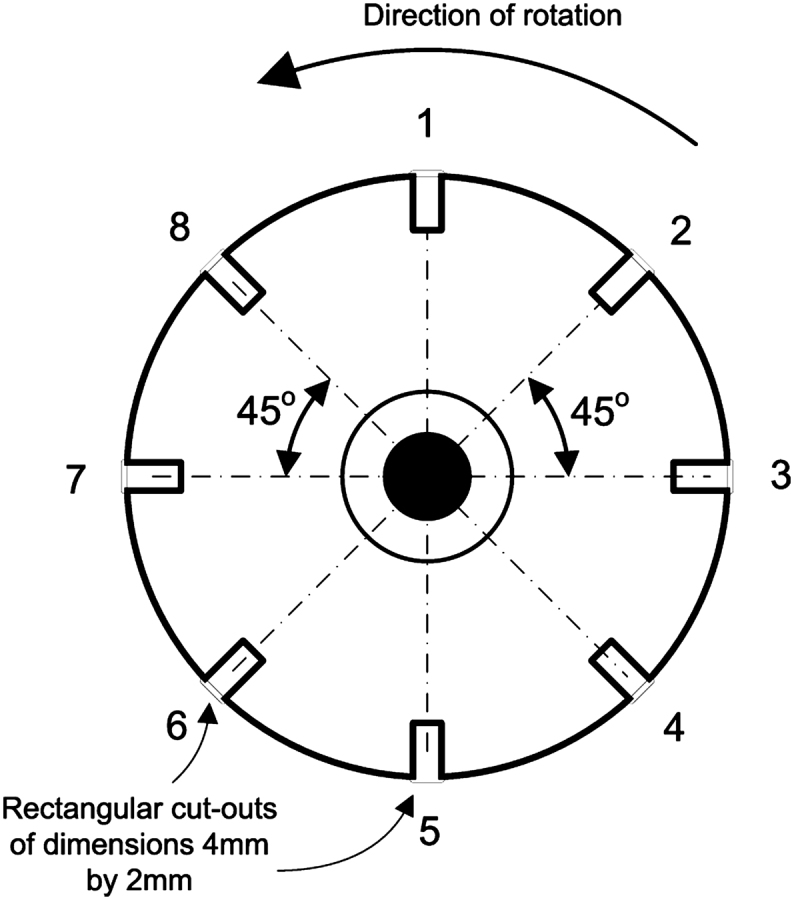
Timing wheel.

An aluminium disc fixed to the rotor (indicated as ‘timing wheel’ on Fig. 1 and shown in detail in Fig. 2) has eight cut-outs equally spaced around the perimeter. These cut-outs are detected by an optical transmission sensor (Honeywell HOA2001) mounted on the back of the Dural^®^ rotor support. The signal from this sensor is used to synchronise the imaging system (see Section 2.3).

**Fig. 3:**
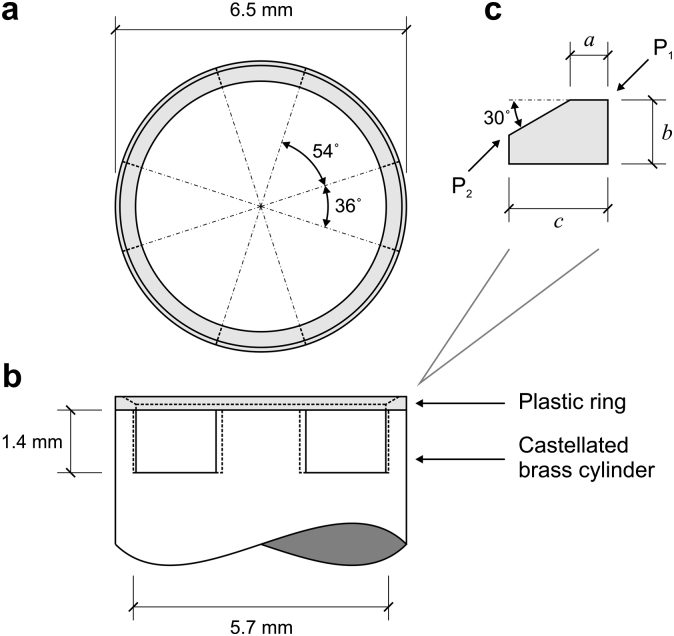
Lens support detail; (a) plan, (b) elevation and (c) Delrin^®^ ring cross-section. Support ring dimensions determined from lateral calibration images collected during the test programme are *a* = 0.2 mm and *c* = 0.6164 mm. Data obtained from separate measurements made after completion of the test programme are *a* = 0.2105 mm, *b* = 0.3573 mm and *c* in the range 0.5201 mm–0.5841 mm.

The lens specimen is placed on an interchangeable lens support located centrally on the top of the rotor shaft. Supports are accurately located on the rotor by a central pin and secured with two opposing grub screws. Suitable dimensions for the support were determined on a trial-and-error basis. A support design that was found to provide a good compromise between lens visibility and lens stability is shown in Fig. 3. In this design, the lens is supported on a ring machined from a thermoplastic material known as Delrin^®^. (The ring was fashioned from plastic, rather than metal, to reduce the risk of damaging the lens while it is being positioned.) The surface of the ring is inclined at 30° so that it matches, approximately, the local slope of the posterior surface of the lens. The plastic ring is glued to a castellated brass cylinder. It was found that a well-positioned lens could be spun at over 2000 rpm without falling off this support.

**Fig. 4:**
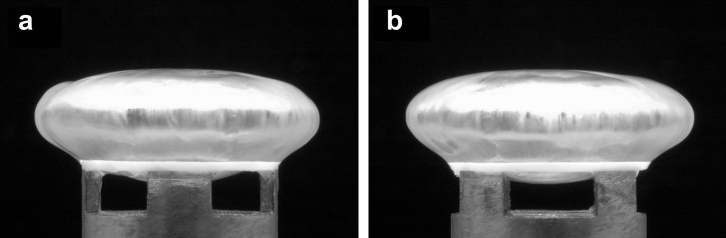
De-capsulated 33-year lens at a rotational speed of 1000 rpm. Figs. (a) and (b) show the lens at a relative angular orientation of 45°.

By careful angular alignment of the timing wheel with respect to the castellations in the lens support, it is possible to obtain images for which in half of the cases the lower surface of the lens is visible and in the other half the surface of the lens in the neighbourhood of the support ring is visible. Two images of the 33-year lens when aligned in this way are shown in Fig. 4.

**Fig. 5:**
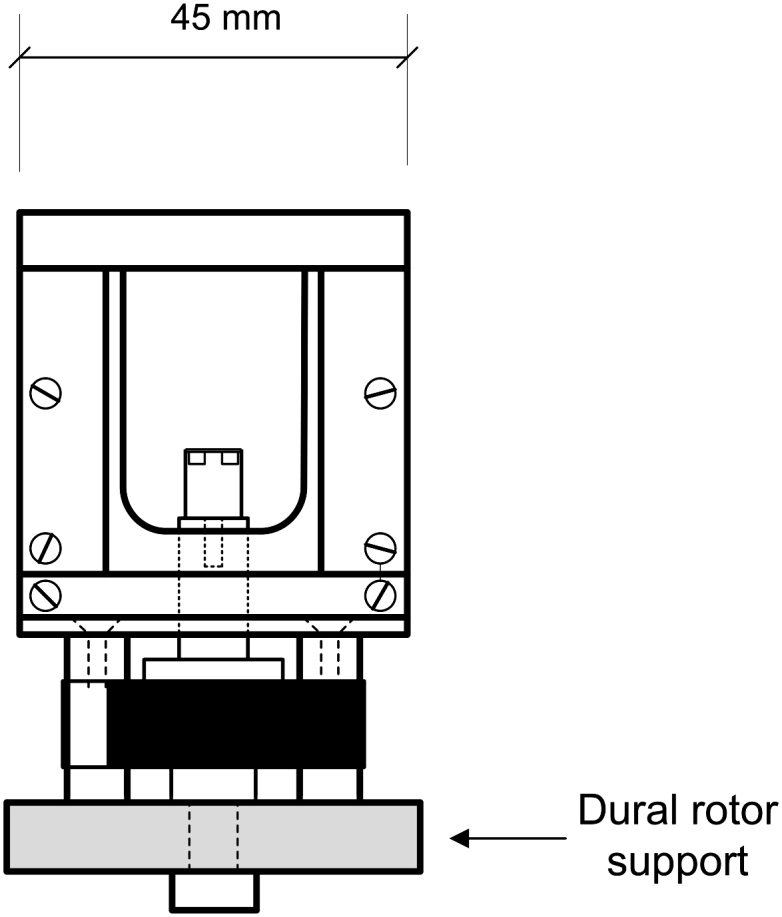
Front view of the lens box.

The lens support is enclosed in a removable PMMA (Perspex^®^) lens box. This box contains the lens should it fall off the support during the test. It also serves the useful purpose of allowing a humid environment to be maintained for the lens; this humid environment minimises drying of the lens during the test. The front of the box (see Fig. 5) has an open window that is sealed with a removable microscope cover slip (thickness #1, 0.13–0.16 mm). The lens is imaged through this window using the system described in Section 2.2. The box has a removable clear lid to allow illumination of the lens from above. The floor of the box is covered in aluminium foil to reflect light onto the underside of the specimen. A piece of black card is mounted at the rear of the box to provide a dark background for the photographs. It is angled downwards to avoid direct illumination from the flash. A pool of water on the floor of the box and damp filter paper on the sides keep the box humid. The latter also enhances illumination of the lens.

### 2.2 Imaging system

Images of the lens profiles are acquired using a Nikon D70 digital single-lens 6 megapixel reflex camera fitted with a Nikon Micro-Nikkor 55 mm macro lens and three Nikon PK-13 extension rings. The camera is mounted on a two-axis travelling microscope stand that has been modified to allow adjustment in the third axis and tilt. To set the system up, the macro lens is adjusted to its most extended position, and the whole camera is moved (via the travelling microscope micrometer screw) so the focal plane coincides with the axis of the support. This gives an image magnification of 1.95, and a resolution of about 4 μm per pixel. The camera is typically used at aperture f/22 with a digital ISO of 400.

The camera is controlled from a laptop computer via a USB connection. A custom program, *LensCam*, controls the camera. It allows the camera settings to be adjusted remotely and for batches of photographs to be captured when initiated by the user. *LensCam* is also used to download the images from the camera once the full test sequence on a lens is complete, to minimise delays during the tests. It assigns systematic names to the resulting files.

The lens is illuminated by a flashgun (Nikon Speedlight SB-800) positioned directly above the lens box. It is used at its shortest flash duration setting (manufacturer’s specification 24 μs). The flashgun is triggered by the timing system described below so that it is synchronised with the angular position of the lens. The camera is set to a long exposure (typically 1.3 s) to ensure the shutter is open when the flash is triggered at the lowest rotation speed employed in the tests. During testing, the room is kept dark and a shroud is placed over the camera and rig to ensure that the image is formed only during the period of illumination from the flash.

### 2.3 Timing system

A custom timing system based on a PIC16F876 microcontroller chip (referred to below as the PIC) is used to synchronise the flash with the rotor position. This system receives the flash signal from the camera when its shutter opens; it then relays this signal to the flashgun at the precise time needed to illuminate the lens when it is at the desired angular orientation. The PIC monitors the position of the lens by counting rising edge signals from the timing wheel sensor. This count is reset every rotation by the rising edge signal from the reset flywheel to ensure that any spurious signals cannot cause a persistent error in the calculated position. An image is captured at each of the 8 angular orientations determined by the angular positions of the slots on the timing wheel. Each time the flash is triggered the target position is incremented by one, with Position 1 following Position 8, so a batch of eight images will consist of one at each timing wheel position.

There was found to be a system delay, *δ*_1_, of about 70 μs between sending the flash signal to the flashgun and the actual illumination of the lens. If this is not accounted for, it would lead to slightly different angular imaging positions at different speeds of rotation. To avoid this angular error, when the timing system is set to trigger at Position *N*, it counts the time, *δ*_2_, between the arrival of the signals for Position *N*–2 and Position *N*–1. The flash is triggered at time *t_N_*_−1_ + *δ*_2_ − *δ*_1_ where *t_N_*_−1_ is the time of arrival of the signal from Position *N*−1. The illumination therefore coincides precisely with the arrival of the signal for Position *N*. For speeds below about 100 rpm this procedure becomes inaccurate. Since at these low speeds any system delays are not problematic, to image the lens at Position *N* the timing system simply triggers the flash when the signal for Position *N* is received.

### 2.4 Test procedure

Human lenses are received from the Bristol Eye Bank, UK, where the iris, ciliary body, zonules, and lens are removed as a unit from the eye globe. (Appropriate ethical permissions were obtained to cover the use of this tissue for the purpose of this research.) The lenses are transported (by courier) in Sigma Megacell Minimum Essential Medium Eagle (M4067) with Sigma Antibiotic–Antimycotic Stabilized (A5955) at ambient temperature. In the testing laboratory, the lenses are kept in this same medium and at room temperature (typically 21–22 °C). Testing commences as soon as practical after the lenses arrive at Oxford University, UK. Immediately prior to testing, any extraneous tissue (e.g. zonule) is removed from the lens under a dissecting microscope.

To conduct a test, the lens is carefully placed on the lens support with its anterior surface uppermost. It is manipulated, using ophthalmic spears, to ensure that it is accurately mounted with the lens axis on the rotor axis. Mounting of the lens is checked by eye by observing the lens as the rotor is slowly rotated by hand. The lens is then dabbed dry using ophthalmic spears. The lens box is then placed over the lens and the flashgun and shroud are moved into position.

All lenses were subjected to the primary test sequence shown in Table 1. Initially, Test A_ref_, the speed is set to 70 rpm and a set of 8 images are captured. These images are regarded as reference images in the sense that the lens is rotating sufficiently slowly for centripetal forces to be negligible. The speed is then increased to 700 rpm and a further set of 8 images is obtained (Test A). Another set of reference images at 70 rpm is then obtained (Test B_ref_). The speed is then increased to 1000 rpm and a further set of images is obtained (Test B). Finally the speed is reduced to 70 rpm and another set of reference images is captured (Test C_ref_). Some of the lenses investigated in the test programme (although not including the 33-year lens test described in this paper) were subjected to a further loading cycle to 1400 rpm.

Once this initial testing sequence has been completed, the lens is removed from the support and returned to the medium in a Petri dish. The lens capsule is then removed under a dissecting microscope with two pairs of forceps. One pair takes hold of the anterior capsule away from the equator and the pole; this pair is raised so the capsule pulls away from the lens and the second pair takes hold of the stretched capsule nearby. Both pairs of forceps are then used to tear the capsule and pull it from the lens, generally without making contact with the exposed lens substance. The de-capsulated lens is then returned to the lens mount where it is again centred on the axis of rotation. The primary test sequence specified in Table 1 is then repeated on the de-capsulated lens. In most cases, the de-capsulated lenses were then subjected to a further, secondary, test sequence. The standard approach adopted in the experiment is to use the data from the primary test sequence to determine values of lens stiffness. Data from the secondary tests are available for studies on the possible effects of water evaporation during the test and time-dependent deformations of the lens.

Calibration of the system is achieved by collecting a set of 8 images of a ball bearing of known dimensions placed on the lens support. A ball bearing of diameter 7.93 mm is used for this purpose.

### 2.5 Summary of the procedure for inferring lens stiffness parameters

The initial test sequence provides data on the deformations induced in the intact lens. However, the results of this test sequence cannot be used straightforwardly to determine values of stiffness for the lens substance. Any analytical or numerical model of the performance of the intact spinning lens to process the experimental data would need to include separate constitutive models for the capsule and the lens substance. Attempts to infer details on the stiffness of the lens substance will therefore be poorly conditioned as a consequence of uncertainties in the influence of the capsule on the overall lens deformations (e.g. see Burd et al., 2006). In view of this, attempts to infer values of lens stiffness parameters are confined to the results of the de-capsulated lens tests.

Calculations of lens stiffness are based on the standard approach of using lens outlines captured in Test B for the de-capsulated lens. At the rotational speed employed in this test (1000 rpm) the deformations induced in the lens are within the range of the displacements that develop during accommodation. (It is also noted that this rotational speed is the same as the one adopted by Fisher, 1971). Stiffness parameters are determined by seeking a best fit between the outline of the lens computed using a finite element model and the target outline determined from the images captured in Test B. The geometry used to generate the finite element mesh is based on the combined set of 16 images collected in Tests B_ref_ and C_ref_.

### 2.6 Image processing

To analyse the data obtained from the test it is necessary to quantify the lens outline at desired stages in the test sequence. This is achieved using a customised process, based on MATLAB^®^, to determine the edges of the lens from the images obtained during the test. In most cases this process could run in a fully automated manner. Occasionally, however, debris on the lens or support is found to interfere with the process. In these cases, the images are edited by hand and the image processing procedures are repeated.

Initially, each image is converted to grey scale, and image correlation is performed between the support regions of the images from Tests B_ref_ and B with the corresponding image from Test C_ref_ so that any small camera movement between the corresponding image sets can be detected and then corrected.

Each grey-scale image is filtered to give an approximate vector-valued gradient of intensity at each pixel. Certain features of the lens support, such as the sides, are identified by finding the first or last substantial peak in the appropriate component of the gradient within a relevant band of pixels. These features are used to identify the axis of rotation, the location of the top of the plastic lens support ring, and the region of the image to search for the lower parts of the lens that are visible through the castellations on the lens support.

An origin is selected at the intersection of the axis of rotation with the plane of the top of the plastic lens support ring. The distance from the origin to the outside of the lens is then sought along rays spaced at angular increments of *π*/1000 radians. The intensity gradient is calculated at finely spaced points along each ray using bi-cubic interpolation from the values at the surrounding 16 pixels. The outside of the lens is taken to be the location of the last substantial peak in the gradient component orthogonal to the lens outline. The process begins with vertical rays. The orthogonal direction to the lens outline is assumed to be vertical for vertical, or near vertical, rays. For rays at other angles, the orthogonal direction is estimated by extrapolation from the geometry of nearby points on the lens outline that have already been detected.

### 2.7 Fitting cubic splines to the lens and a circle to the calibration image

A smoothed and averaged axisymmetric representation of the lens outline at each stage in the test sequence is determined by fitting (in a least-squares sense) two cubic splines (in polar coordinates) to the set of 8 outlines determined from the images as described in Section 2.6. One spline represents the geometry above the support ring. This spline has a zero slope condition at the axis of rotation and a fixed end point at the top outside corner of the support ring (Point P_1_ in Fig. 3c). It has seven internal knots spaced at equal angular intervals, with the first knot positioned at half of this interval from the axis of rotation and the last knot positioned at one-eighth of the interval from the fixed end point. A second spline represents the outline of the lens below the supporting ring. The spline is fixed at one end to the inside corner of the support ring (Point P_2_ in Fig. 3c) and, at the axis of rotation, the slope is constrained to be zero. The spline has two internal knots. These two splines, together with the internal lens support ring surface between Points P_1_ and P_2_, form the full axisymmetric outline of the lens.

The reference outline (which is needed for the generation of the finite element mesh that is used to infer the lens stiffness parameters from Test B on the de-capsulated lens) is determined as described above, but on the basis of the complete set of 16 lens outlines captured in Tests B_ref_ and C_ref_.

**Fig. 6:**
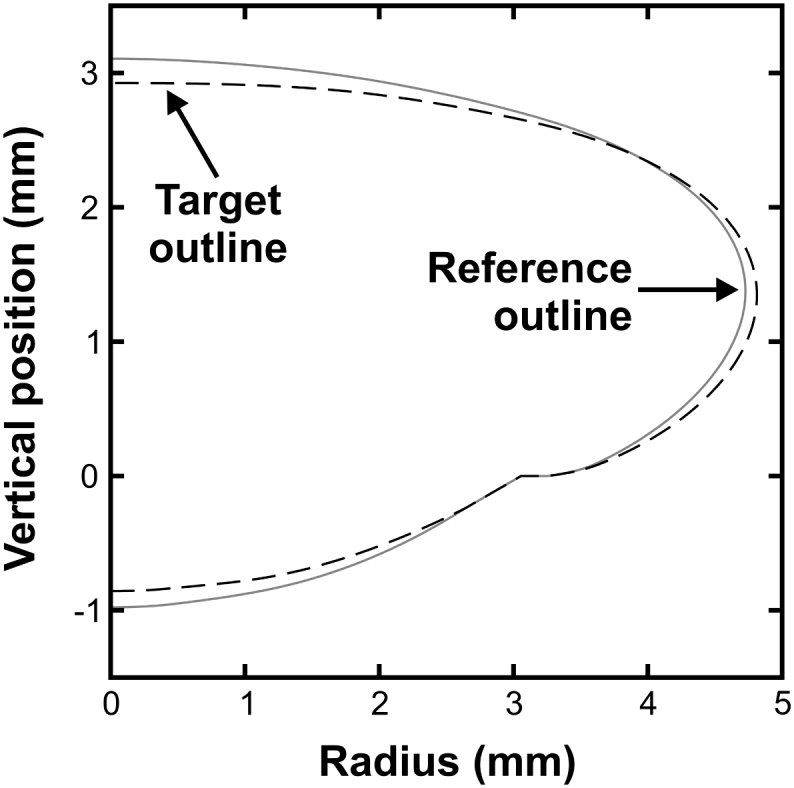
The target outline (dashed line) corresponding to the shape of the lens when spinning at 1000 rpm and the reference lens outline (solid line) corresponding to the shape of the lens when spinning at 70 rpm.

Spline representations of the lens outlines for the 33-year de-capsulated lens are plotted in Fig. 6. The target outline refers to the averaged spline representation of the lens geometry in Test B and the reference outline refers to the averaged spline representation for the lens geometry from the images collected in Tests B_ref_ and C_ref_.

A similar procedure is adopted to analyse the calibration images of the ball bearing, but in this case, a circle is fitted to the outline points identified in each image. The average diameter in pixel units of these 8 circles is compared to the measured diameter of the ball bearing to determine the scale factor (mm/pixel) that is used to calibrate the observed lens outline geometries.

### 2.8 Inferring lens stiffness parameters

Lens outlines determined using the spinning lens test cannot be used directly to establish the nature of the spatial variation of stiffness in the lens. Instead, it is necessary to assume a spatial variation function (SVF) for the stiffness in parametric form. To quantify the lens stiffness profile, the parameters in the SVF are optimised by minimising the error between the target outline and the lens outline computed in a finite element analysis of the spinning lens in which the SVF is incorporated.

**Fig. 7:**
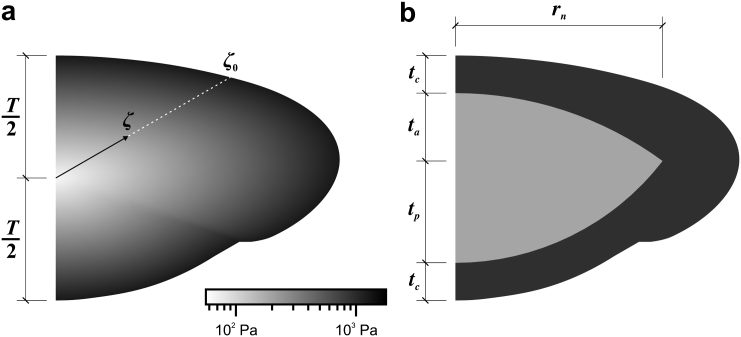
Spatial Variation Functions. Figure (a) illustrates Model E and Figure (b) illustrates Model D. The grey scale shown is based on the optimised stiffness parameters for the 33-year lens (see Fig. 11). The dimension *T* is the axial thickness of the lens. The dimensions adopted for Model D model are: *r_n_* = 3.45 mm, *t_a_* = 1.132 mm, *t_p_* = 1.698 mm. The dimension *t_c_* is determined by the total axial thickness, *T*, of the particular lens being modelled.

Three SVF’s were adopted. In one of these, termed ‘Model H’, the lens substance is assumed to be homogeneous (i.e. the shear modulus does not vary spatially). Although this model does not represent the non-homogeneity that is known to exist in the lens, it is adopted to provide an overall check on the values of shear modulus obtained from the other two (more detailed) models. In another model, termed ‘Model E’, the shear modulus varies exponentially with distance from the centre of the lens. In the third model, termed ‘Model D’, the nucleus and cortex are represented as distinct entities; this provides a two-parameter model defined in terms of the nucleus shear modulus, *G_N_*, and the cortex shear modulus *G_C_*. Models E and D, shown on the reference outline for the 33-year lens, are illustrated in Fig. 7. The grey scales on this figure correspond to the optimised stiffness parameters determined for this particular lens.

Model E is relatively easy to define. The centre of the lens is assumed to be the mid-point between the anterior and posterior poles on the optical axis. The shear modulus, *G*, at any point in the lens is defined by:

(1) $$G=\frac{\alpha \exp\left(\beta \zeta \right)}{\zeta_{0}}$$

where *ζ* is the distance of the point from the mid-point of the lens, *ζ_o_* is the distance to the lens outline along a ray passing through the point (see Fig. 7a) and *α, β* are the two parameters defining the model.

Model D is less straightforward to define as a consequence of need to specify a shape for the nucleus. To determine an appropriate nucleus geometry, a survey of several measurements of nucleus dimension using different techniques was made (Brown, 1973; Dubbelman et al., 2003; Hermans et al., 2007; Kasthurirangan et al., 2008; Sweeney and Truscott, 1998; Ayaki et al., 1993; Gullapalli et al., 1995). It is clear (e.g. see Augusteyn, 2010) that it is not possible to determine a unique specification for nucleus geometry from the available literature. The following model was therefore adopted on the basis that it is thought to provide a representation of the nucleus geometry that is consistent with broad range of the available data. The shape of the nucleus was formed from two arc segments (or spherical caps in 3D), following Burd et al. (2002). The dimensions of the equatorial radius, *r_n_* and polar thickness (*t_a_* + *t_p_*) of the nucleus were taken from the clearest class of cataractous nuclei in Gullapalli et al. (1995). (These data were selected because they are towards the centre of the whole collection of measurements, they represented a large set of samples, and the definition of the nucleus adopted by Gullapalli et al. (1995) seems to be consistent with the notion of the nucleus as a mechanically distinct region.) The position of the nucleus equator was specified by 3*t_a_* = 2*t_p_*. Numerical values of the various dimensions determined in this way are given in the caption of Fig. 7.

The finite element inverse analysis is based on a non-linear hyperelastic approach (Bonet and Wood, 1997) using the program *Oxfem_hyperelastic* that has been developed at Oxford University for the study of problems in ophthalmic mechanics. The lens is represented by a neo-Hookean constitutive model with the strain energy function, ψ(**C**), given by:

(2) $$\psi \left(C\right)=\frac{\mu }{2}\left(tr\left[J^{-2/3}C\right]-3\right)+\frac{\kappa }{2}{\left(J-1\right)}^{2}$$

where **C** is the right Cauchy-Green tensor, *J*^2^ = det **C** and *μ* and *κ* are material constants.

For the case where the deformations are small, the behaviour of the neo-Hookean model corresponds to the behaviour of a conventional linear elastic material for which *G* = *μ* and *K* = *κ* (where *G* and *K* are the shear modulus and bulk modulus, respectively, as adopted in conventional linear elasticity). In this paper, therefore, the parameter *μ* is referred to as shear modulus and is given the symbol *G* in recognition of the close link that it has with the shear modulus as defined in linear elasticity. In conducting the finite element optimisation analysis, the value of *κ* is set to *κ* = 100 *μ*. In terms of conventional linear elasticity, this corresponds to a Poisson’s ratio of 0.495.

The finite element analysis is based on a conventional displacement approach, formulated in axisymmetry. The formulation is based on the solution to the weak form:

(3) $$0=2\pi \left(\int_{A}\delta \Psi \left(C\right)RdA-\int_{a}b\text{.}\delta urda\right)$$

where *A* is the area occupied by the axisymmetric cross-section of the lens in the material configuration and *a* is the area occupied by the axisymmetric cross-section in the spatial configuration. Similarly, *R* and *r* are radial coordinates in the material and spatial configurations respectively. The displacement at a material point is defined by the vector **u** and *δ*(·) is the variational operator. The body force vector, **b**, has one non-zero component, *br*, in the radial direction given by:

(4) $$b_{r}=\rho r\omega^{2}$$

where *ρ* is density and *ω* is angular velocity. The analyses are based on $\rho =1058.98\;\text{kg}/{\text{m}}^{3}$. This value is adopted on the basis of the regression given in Burd et al. (2002) (after Bellows, 1944) for a lens of age 40 years. (Although the density of the lens is thought to vary slightly with age, this age dependency was not considered in the optimisation analysis described in this paper).

Gravity loads are excluded from the analysis. Although the gravitational forces applied to the lens are sufficient to induce visible deformations in the lens in the neighbourhood of the support, it should be noted that the analysis of the test is concerned with the change in lens outline as a consequence of the centripetal forces induced by spinning. It is assumed that this change in lens outline is unaffected by the presence of gravitational loading.

**Fig. 8:**
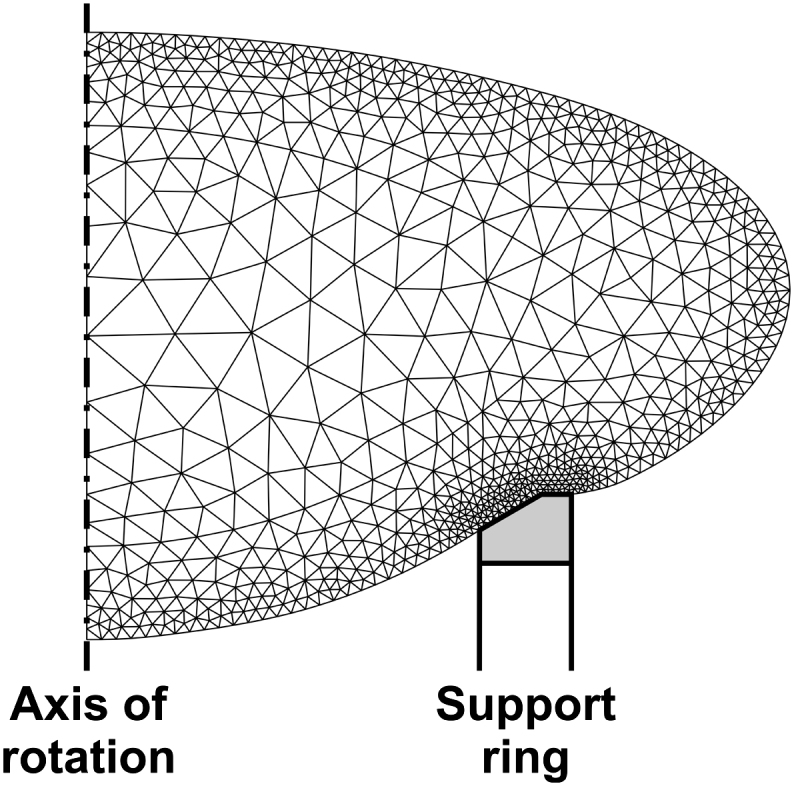
Reference finite element mesh for the 33-year lens.

The finite element mesh used to conduct the inverse analysis is generated on the reference lens outline. The dimensions of the lens support ring adopted in the finite element mesh generation determined from calibration images obtained during the test programme are *a* = 0.2 mm and *c* = 0.6164 mm (see Fig. 3c). Mesh generation is conducted using routines available for MATLAB^®^ (Mesh2D written by D. Engwirda). The mesh consists of fifteen-noded triangles with a thirteen-point Gauss integration scheme (Cowper, 1973). The mesh is generated to include an internal zone that corresponds to the assumed geometry of the nucleus. Meshes typically consist of about 1500 elements and 12000 nodes. The mesh used for the analysis of the 33-year lens is shown in Fig. 8.

A key issue to be addressed in the finite element analysis is the choice of model to represent the contact mechanics and the interface of the lens and the ring support. The simplest approach, from a computational perspective, would be to treat the support as fully-adhesive. In this case, the nodes in contact with the support are prescribed as having zero displacement. An alternative approach is to treat the contact between the lens and support ring as being both frictionless and adhesionless. In this case the lens is free to slide over the surface of the support. In the following discussion the fully-adhesive condition is referred to as ‘fully-fixed’ and the frictionless/adhesionless condition is referred to as ‘smooth’.

The fully-fixed and smooth contact conditions relate to two extremes of behaviour. It seems likely that the actual behaviour of the contact will fall between these two extremes, i.e. some slippage at the support may occur but, at the same time, effects due to surface tension, adhesion and friction will generate inward-acting shear stresses that act to limit the relative movement between lens and support.

The fully-fixed condition is implemented, straightforwardly, in the finite element analysis. The smooth support condition requires special consideration; this support condition is implemented in *Oxfem_hyperelastic* as follows. An initial estimate is made on the nodes in the finite element mesh that, at the end of the analysis, will be in contact with the inclined face of the support; this group of nodes is termed Group 1. An estimate is also made of the nodes that, at the end of the analysis, will be in contact with the horizontal surface of the support; this group of nodes is termed Group 2. A finite element analysis is then conducted in which the nodes in Group 1 are prescribed to lie on a straight line that coincides with the inclined face of the support. Similarly, nodes in Group 2 are prescribed to lie on a second straight line that coincides with the horizontal face of the lens support. If the initial estimate of nodes within each group is correct, then at the end of the analysis (when the desired radial body force has been applied), all of the Group 1 nodes will lie on the inclined face of the support and all of Group 2 nodes will lie on the horizontal face of the support. Alternatively, if the initial estimate of nodes within Groups 1 and 2 is found to be incorrect, an automated procedure is adopted to adjust the nodes within each group and then to repeat the finite element analysis until a correct configuration is obtained. These numerical procedures ensure that nodal forces parallel to the support surface are zero (within the tolerance of the analysis) thereby modelling a smooth contact.

**Fig. 9:**
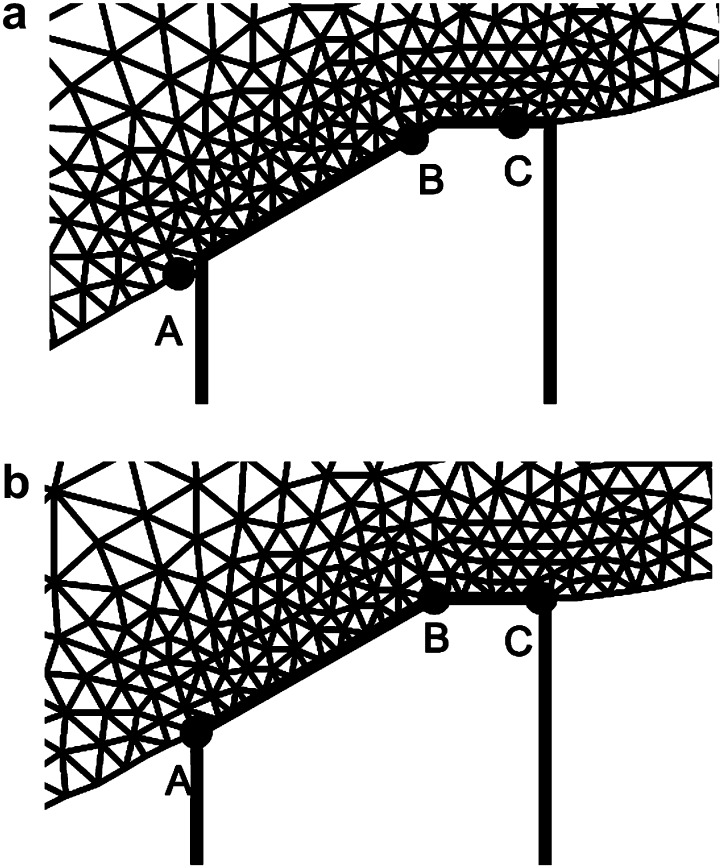
Movement of the lens relative to the support for the 33-year lens, smooth support case. The Group 1 nodes lie between Points A and B and Group 2 nodes lie between Points B and C. The location of these nodes is shown in Fig. (a) at the start of the analysis, corresponding to a stationary lens, and in Fig. (b) where the lens is spinning at 1000 rpm. In Fig. (b), the points A, B and C are seen to coincide correctly with the geometry of the support.

The mesh for the 33-year lens in the neighbourhood of the support for the smooth analysis is shown in Fig. 9a at the start of the analysis (i.e. when the applied radial body force is zero) and in Fig. 9b at the end of the analysis (when radial body forces corresponding to spinning at 1000 rpm are applied). These figures illustrate the computed movement of the lens relative to the support for the smooth contact case.

To compute the lens stiffness parameters, a set of trial SVF parameters are initially assumed and a forward axisymmetic finite element analysis is used to compute the corresponding deformed lens outline. The SVF parameters are then optimised, using the Nelder and Mead (1965) algorithm, to minimise the parameter *A_i_*, which is the area enclosed between the computed and target outlines. For most of the human lenses that have been tested in the current test programme (including the 33-year lens adopted in this paper to illustrate the experimental method) the smooth support condition provided a superior fit (i.e. a smaller value of *A_i_*) than the fully-fixed condition. On this basis all of the data reported below are based on the smooth support condition.

## 3 Results

**Fig. 10:**
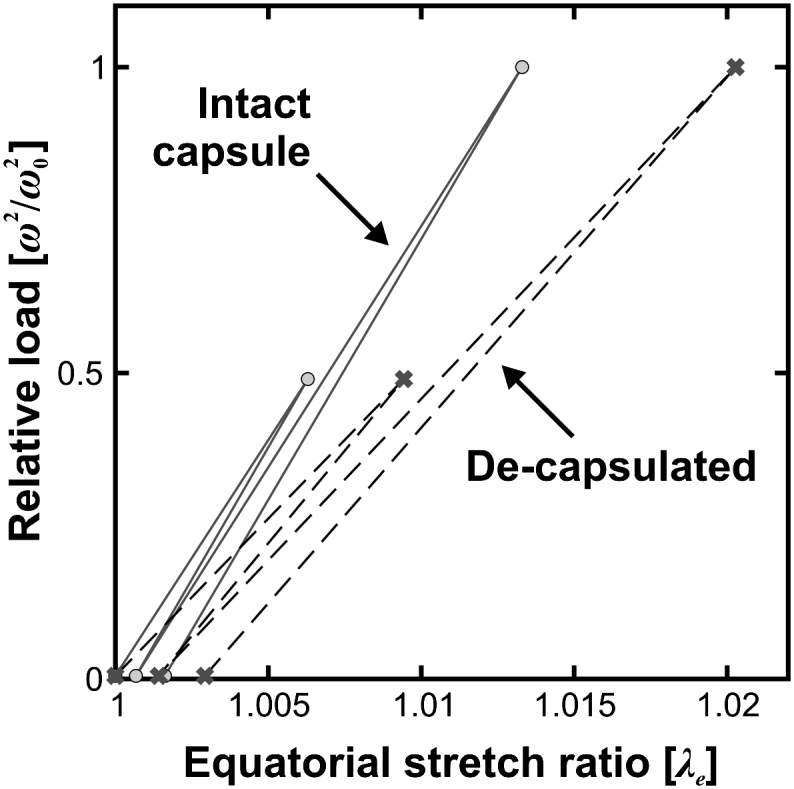
Equatorial stretch ratio, *λ_e_* for the 33-year lens for the test sequence given in Table 1.

The spline determined from Test A_ref_ is used to calculate a reference lens equator diameter at the start of each test sequence. The lens equator diameter is also calculated from the splines obtained for the outlines captured in the subsequent tests in each sequence. The equatorial stretch ratio, *λ_e_*, is defined as:

(5) $$\lambda_{e}=\frac{\text{current}\;\text{lens}\;\text{diameter}}{\text{reference}\;\text{lens}\;\text{diameter}\;\text{at}\;\text{start}\;\text{of}\;\text{test}\;\text{sequence}}$$

where ‘current lens diameter’ refers to the diameter deduced from a spline that is fitted to the images from one of Test A, B_ref_, B or C_ref_. Values of *λ_e_* determined in this way, for the intact 33-year lens and also for the lens when it has been de-capsulated, are shown in Fig. 10. The vertical axis is ‘relative load’ which is defined in terms of the current rotational speed, *ω*, and a reference rotational speed, *ω*_o_, where *ω*_o_ corresponds to 1000 rpm. It is noticeable, particularly for the de-capsulated lens, that the lens diameter does not fully recover when the radial body forces are removed. This is thought to be associated with time-dependent deformations in the lens and the effects of preconditioning (e.g. Fung, 1993). This tendency of the lens to exhibit irrecoverable deformations is typical of the results of the test program. The process used to determine the stiffness parameters (in which the reference profile for the finite element analysis of Test B is based on the combined images from B_ref_ and C_ref_) was designed to minimise, as far as possible, the influence of these time-dependent mechanisms.

The slope of the loading and unloading lines in Fig. 10 provides a qualitative indication of the stiffness of the lens. It is noticeable that the slope of the de-capsulated lens is some 30% lower than that of the intact lens. This difference in stiffness was consistently observed in the test programme (although with some variation in the magnitude of the effect). This observation has a particular significance. In the procedure described in Fisher (1971), the lenses were generally tested with their capsule intact whereas the method employed by Fisher to determine the lens stiffness from the test data was based on the assumption that the structural action of the capsule was negligible. The results in Fig. 10 demonstrate the desirability of conducting the spinning lens test with the capsule removed so that the mechanical influence of the capsule is excluded from subsequent data processing.

**Fig. 11:**
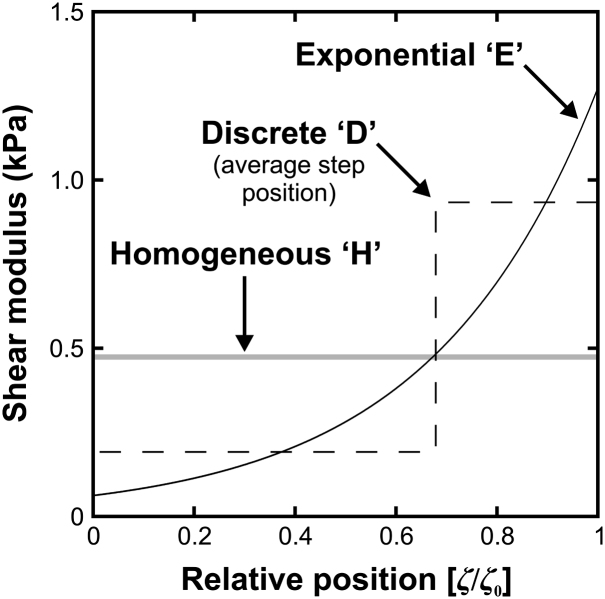
Optimised stiffness data for Models H, E and D. These data are plotted in terms of relative position, *p_r_*. The shape of interface between the nucleus and cortex for Model D means that the interface does not correspond to a single value of *p_r_*. For illustrative purposes, the interface is plotted in the figure at an average value of *p_r_*. Shear modulus for Model H is 0.47 kPa. Parameters for Model E are *α* = 0.062 kPa and *β* = 3.02. Parameters for Model D are *G_N_* = 0.19 kPa and *G_C_* = 0.93 kPa.

The SVF profiles determined from the optimisation procedure for the 33-year lens are shown in Fig. 11. In these plots the relative position, *p_r_*, is defined by:

(6) $$p_{r}=\frac{\zeta }{\zeta_{o}}$$

where *ζ* and *ζ_o_* are defined in Fig. 7a. The profiles appear reasonably consistent with each other in the sense that Models E and D both indicate a nucleus that is substantially less stiff than the cortex. The Model H result provides a value of stiffness that is intermediate, as expected, between the maximum and minimum values of the other two profiles.

The quality of the fit between the optimised finite element model and the target outline may be quantified in terms of the error parameter *γ_E_* where:

(7) $$\gamma_{E}=\frac{A_{i}}{A_{o}}$$

and *A_o_* is the absolute value of the area enclosed between the target and reference outlines. For the 33-year lens, *A_o_* = 0.789 mm^2^ and the values of *γ_E_* computed at the end of the optimisation process are 0.25, 0.075 and 0.080 for Models H, D, and E respectively. These data suggest that Models D and E provide a fit of roughly similar quality and that the quality of this fit is superior to that provided by Model H.

## 4 Analysis of experimental procedures

### 4.1 Sensitivity analysis

**Fig. 12:**
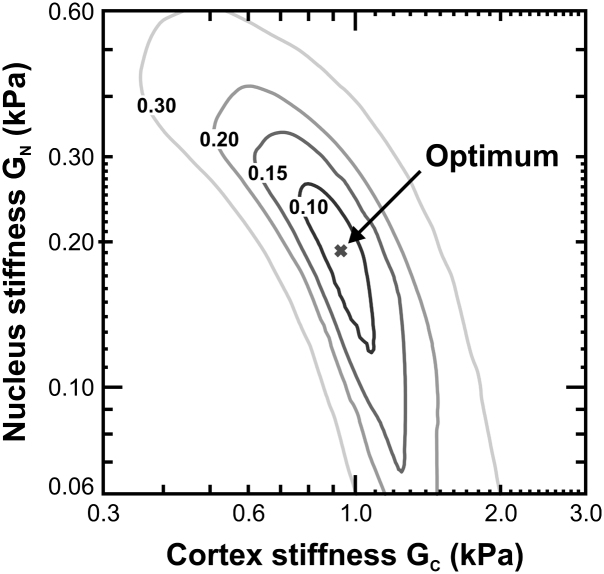
Contours of the error parameter *γ_E_*. (Note that stiffness data are plotted on a logarithmic scale).

An assessment has been undertaken, for Model D, on the influence of variations of *G_N_* and *G_C_* on the value of the computed error parameter *γ_E_*. To conduct this assessment, a set of finite element analyses of the spinning lens test (1000 rpm) were conducted with values of *G_N_* and *G_C_* varied about the optimised values (as given in the caption of Fig. 11). Computed contours of *γ_E_* are shown in Fig. 12.

Fig. 12 demonstrates that *γ_E_* is sensitive to changes in both *G_N_* and *G_C_*. However, it is rather less sensitive to changes in nucleus stiffness than cortex stiffness. This is principally a consequence of the fact that, in this case, the nucleus is substantially less stiff than the cortex. For cases where the nucleus and cortex shear modulus values are similar, then the value of *γ_E_* is found to be rather more sensitive to changes in *G_N_*.

The observation that *γ_E_* depends on both *G_N_* and *G_C_* (although with different sensitivities) provides support for the utility of the inverse analysis procedure that is adopted to determine the stiffness parameters from the test data.

### 4.2 Evaporation of water from the lens during the test

During testing, the lens is enclosed in a humid environment. However, it seems plausible that the lens could experience significant moisture loss during the test sequence. This, in turn, could influence the computed stiffness data.

An attempt has been made to quantify the extent of moisture loss experienced by the 33-year lens (for the case where the lens is de-capsulated) by analysing the images that were obtained from a secondary test sequence (conducted after completion of the primary sequence given in Table 1). Details on the secondary test sequence are given in Table 2.

It is assumed that any evaporation of water during the test will cause a reduction in the volume of the lens. To investigate whether any volume changes occur, the lens volume was computed for each set of reference images (i.e. images collected at 70 rpm) in the primary and secondary test sequences. In each case, the lens volume was estimated from a volume integral based on the outline identified for each image (rather than the splines used to construct the reference finite element mesh). In each image the posterior surface of the lens is partially obscured by the support castellations; outlines for these obscured regions were determined from the subsequent photograph in the series (in which the lens position is incremented by 45°). The average of the eight individual volume integrals is used as the estimate of the lens volume.

**Fig. 13:**
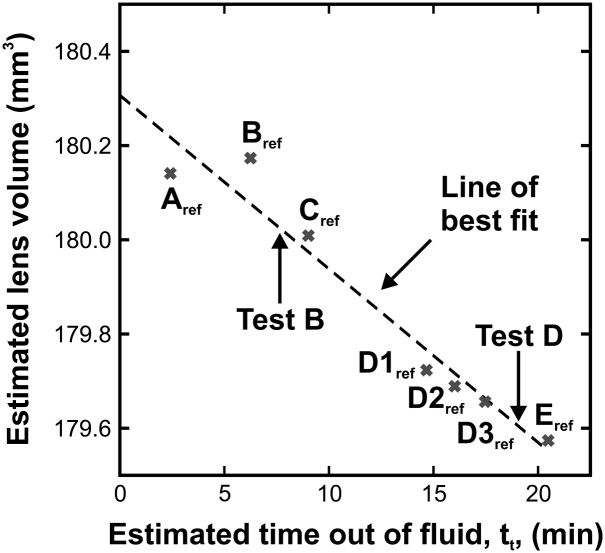
Variation with time of the estimated lens volume for the 33-year de-capsulated lens.

Data on lens volumes computed in this way are plotted on Fig. 13, where the estimated time out of fluid, *t_t_*, is the time between removing the de-capsulated lens from the medium and the average time at which the 8 images in each reference set were collected. The least-squares line through the seven available volume data points has a slope of −0.037 mm^3^ min^−1^ (*R*^2^ = 0.947). Thus at the completion of Test B (8.0 min after placing the lens on the support) the lens is estimated to have lost about 0.3 mm^3^, or 0.16% of its initial volume. (Note that the calculation of lens volume is not exact due to the fact that some of the lens is obscured by the support ring. The volume of lens hidden by the ring is assumed in the analysis to be constant. However, this volume is likely to increase slightly as the test proceeds as a consequence of the observed tendency of the lens to ‘sink’ gradually on the support. The 0.16% figure can therefore be taken as an upper bound on the loss). This estimated amount of volume lost during the extended test sequence seems sufficiently small to justify the assumption that moisture loss does not have a substantial effect on the stiffness data computed at Test B.

The regression line in Fig. 13 suggests that the estimated lens volume at *t_t_* = 0 is 180.3 mm^3^. For a density of $\rho =1058.98\;\text{kg}/{\text{m}}^{3}$ this gives an estimated lens weight of 191 mg. The regression line data in Fig. 3 of Augusteyn (2010) gives the expected weight of a 33-year lens as 195 mg. This agrees well with the above estimate (noting that the estimate of 191 mg does not include the weight of the material removed when the lens was de-capsulated). This comparison provides additional assurance on the calibration and data analysis methods employed in the tests.

As a further check on the possible effect of moisture loss (and also to investigate the repeatability of the measurements), a set of independent stiffness data was determined from the images collected in Test D (see Table 2) on the basis of Model D for the lens. These stiffness data ($G_{N}=0.21\;\text{kPa}$, $G_{C}=1.0\;\text{kPa}$, $\gamma_{E}=0.098$) correspond closely to the stiffness data determined from Test B in the primary test sequence (see Fig. 11). The average time at which the Test D data were collected was *t_t_* = 19.0 min and the average time at which the Test B data were collected was *t_t_* = 7.7 min. The fact that the numerical differences between these two sets of stiffness data are small provides further support for the assumption that the computed stiffness data are insensitive to any evaporation of water that may occur during the test procedure.

## 5 Discussion

This experimental procedure incorporates various improvements over the original version of the test devised by Fisher (1971). The use of a synchronised imaging system, for example, means that the lens can be imaged, systematically, at equally spaced angular orientations. This provides a systematic way of determining averaged outlines for the lens. The imaging system employed in the experiment provides images that appear free of motion blur. This is in contrast to some of the images given in Fisher (1971) that appear to suggest that significant rotational motion of the lens occurred while the photograph was being taken. If Fisher’s results were affected by motion blur, then this might have led to artificially high values of lens equatorial diameter change being deduced from the photographs (particularly for relatively stiff lenses).

Importantly, in the current experiment the stiffness parameters of the lens are based on tests in which the lens is de-capsulated. Removing the lens capsule may induce a certain amount of mechanical disturbance to the outer fibres of the lens. It may also make the lens more susceptible to moisture loss (although estimates given in this paper suggest that that effect of moisture loss is unlikely to be substantial). It should be noted, however, that the test data on de-capsulated lenses have the very considerable advantage that it is not necessary to consider the mechanics of the capsule in any analysis to determine the stiffness parameters for the lens substance.

The finite element optimisation procedures described in this paper are lengthy and laborious. However, they have considerable advantages over approximate methods of the sort employed by Fisher (1971). In particular, this approach provides opportunities for the modelling errors inherent in the analysis procedures to be systematically controlled and minimised. It is recognised, however, that the inverse analysis procedure described in this paper relies on the existence of a parameter with a clearly-defined minimum value with respect to the stiffness parameters. Although the parameter *γ_E_* is seen to exhibit a well-defined minimum (for the Model D case and the 33-year lens), the shape of the error surface is such that the nucleus stiffness is likely to be estimated with less confidence than the cortex stiffness.

The spinning lens test cannot be used to determine a unique functional form for the variation of stiffness within the lens. This suggests that it should be regarded as complementary to other forms of stiffness test in which local measurements are possible. It should be emphasised, however, that the spinning lens test has the very considerable advantage over procedures such as indentation testing in that the internal structure of the lens remains undisturbed by the test procedure.

Stiffness parameters are determined on the basis of lens outlines captured at a rotational speed of 1000 rpm. It would also be possible to determine stiffness parameters from the tests conducted at other speeds. This would provide an additional check on the stiffness data. It might also provide an indication of any non-linearity (if any) in the material behaviour.

The analysis of the test is conducted on the basis that the lens can be represented by an isotropic nearly-incompressible elastic material. On this basis, it is possible to obtain a reasonable fit between the optimised finite element model and the test data. It is plausible, however, that as a consequence of the highly structured nature of the lens an improved fit would be obtained with the use of an anisotropic model in which the principal directions of the model are aligned with the local lens fibre orientations. However, although a model of this sort may prove be useful in the future (as more data on the mechanics of the lens at a lens fibre level become available) it is considered that, at present, there are insufficient data for a robust model of this sort to be devised.

## Figures and Tables

**Table 1:** Primary test sequence.

Test Reference	A_ref_	A	B_ref_	B	C_ref_
Rotational speed (rpm)	70	700	70	1000	70

**Table 2:** Secondary test sequence conducted on the 33-year de-capsulated lens. Test C consisted of maintaining the rotor speed at 1000 rpm while three sets of images – C1, C2 and C3 – were obtained. The rotor speed was then reduced and three further reference images – D1_ref_, D2_ref_ and D3_ref_ – were obtained. A final set of images – D – were then collected; these images were processed to determine lens stiffness parameters on the basis of the reference images D3_ref_ and E_ref_.

Test Reference	C1	C2	C3	D1_ref_	D2_ref_	D3_ref_	D	E_ref_
Rotational speed (rpm)	1000	1000	1000	70	70	70	1000	70
